# Arkansas Medical Association

**Published:** 1877-05-20

**Authors:** 


### ARKANSAS MEDICAL ASSOCIATION

The Medical Association was held at Hot Springs, on the 18th and 19th ult. Dr. W. H. Barry, of Hot Springs, delivered an interesting address.

The members were cordially received and treated with generous hospitality by the citizens and members of the Medical Profession.

Dr. Hazard, editor of the St. Louis Chemical Record, was present by invitation, and addressed the convention.

Resolutions were passed commendatory of the Clinical Record, Virginia Medical Monthly, Southern Medical Record, Philadelphia Medical and Surgical Record, and Chicago Medical Journal and Examiner, for the interest manifested by those journals in that side of the Arkansas medical troubles represented by the Association. Also, in relation to securing a higher standard of medical education, by pledge.

[We are not familiar with all the facts connected with the Arkansas medical troubles alluded to in the above resolution. We disclaim any prejudice against either party; but we are the advocates of truth and justice, and simply oppose hasty and impulsive action in our deliberative bodies. The good of the profession at large requires this, and any other action will not be sanctioned by the true and good men of the profession. We are in favor of a full and impartial hearing in all such controversies before definite or final action is taken. The commission of an error does not necessarily criminate the party committing it, as it may be ignorantly done, but to endorse and perpetuate a wrong, with all the facts before you, would be partisan in spirit and corrupt in principle.]

The following gentlemen were elected to the offices specified for the coming year: J. A. Dibrell. jr., M. D., of Little Rock, President; J. L. H. Sessums, M. D., of Lonoke, First Vice President; T. J. Ried, M. D., of Hot Springs, Second Vice President; J. M. Gist, M. D., of Beebe, Third Vice President; D. H. Dungan, M. D., of Little Rock, Permanent Secretary, and J. M. Pirtle, M. D., of Little Rock. Treasurer.

The Association adjourned to meet at Little Rock, on the fourth Tuesday in April, 1878.

